# The Impact of Trace Elements on Osteoarthritis

**DOI:** 10.3389/fmed.2021.771297

**Published:** 2021-12-23

**Authors:** Guoyong Li, Tao Cheng, Xuefeng Yu

**Affiliations:** ^1^Department of Orthopaedics, The Fourth Affiliated Hospital of Nanchang University, Nanchang, China; ^2^Department of Orthopaedics, Shanghai Jiao Tong University Affiliated Sixth People's Hospital, Shanghai, China

**Keywords:** trace elements, excess, deficiency, role, osteoarthritis

## Abstract

Osteoarthritis (OA) is a progressive degenerative disease characterized by cartilage degradation, synovial inflammation, subchondral sclerosis and osteophyte formation. It has a multifactorial etiology with potential contributions from heredity, endocrine function, abnormal mechanical load and nutrition. Of particular considerations are trace element status. Several trace elements, such as boron and magnesium are essential for normal development of the bone and joint in human. While cadmium correlates with the severity of OA. The present review focuses on the roles of trace elements (boron, cadmium, copper, iron, magnesium, manganese, selenium, zinc) in OA and explores the mechanisms by which they act.

## Introduction

Osteoarthritis (OA) is a prevalent age-related degenerative disease, which is characterized by degeneration of articular cartilage and damage to the other joint tissues (1). The process of OA may begin with some specific parts of joint but ultimately manifests as the whole joint tissue (2). According to the Global Burden of Disease Study 2017, the prevalence of OA is more than three hundred million with the incidence nearly to fifteen million (3). The incidence of OA may continue to accelerate owing to the prevalence of obesity and the increase of life expectancy worldwide (4). Progressive pain and stiffness of the joints, induced by OA, result in poor quality of life (5) and shorten life expectancy of the elderly worldwide (6). It is therefore of global importance to treat this debilitating disease. However, to date, no known ideal method for the treatment of OA is available (7). Consequently, OA has become one of the major health problems for the global aging of the population (8).

OA involves oxidative stress, articular cartilage degradation, synovitis and their interplay (9). During the progression of OA, there are more protein catabolism than anabolism, which causes irreversible changes in the articular cartilage structure. As a result, proteoglycans and collagen fibers were degraded (10). The complex pathogenesis of OA includes the interaction of many factors ranging from genetic predisposition to gene expression changes through mechanical load alteration experienced by articular cartilage (11). Disorders in the molecular repertoires can induce the degradation of articular cartilage and trigger the onset of OA (12).

**Figure 1:**
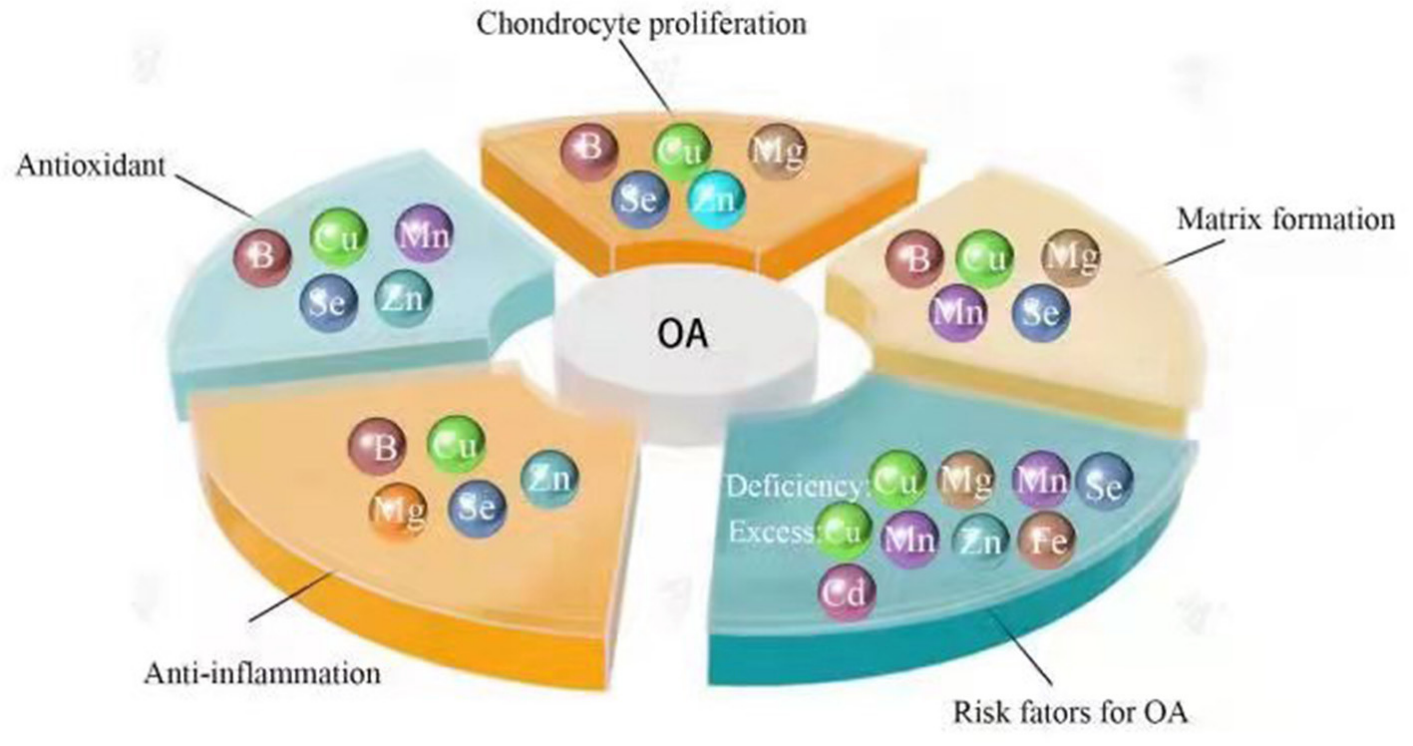
The effects of trace elements on OA. B, boron; Cd, cadmium; Cu, copper; Fe, iron; Mg, magnesium; Mn, manganese; Se, selenium; Zn, zinc. Trace elements such as boron and selenium have anti-inflammatory and antioxidant effects, increasing cartilage matrix formation and enhancing chondrocyte proliferation, resulting in preventing and treating OA. However, excessive or insufficient trace elements like copper are risk factors for osteoarthritis.

OA is viewed as a multifactorial disorder with predisposition factors such as heredity, endocrine disorder, age, gender, joint biomechanics, abnormal mechanical load, weight gain, history of joint surgery and nutrition (13–19). During the long-term remodeling, the joint adapts to specific contents of trace elements. Any changes in the trace element level exhibiting in the excess or deficiency in the joint may impair the function of the joint system and predispose the joint to OA (20). The effects of trace elements mainly depend on their type and content. Some trace elements such as boron and magnesium can prevent and treat OA (21, 22). However, exposure to toxic elements like cadmium may result in the onset and progression of OA (23). The positive or negative effects of trace elements are decided by very narrow concentration range. Trace elements such as copper are beneficial to bones and joints at appropriate concentrations (24). But too low or too high concentrations may trigger OA (25, 26) (Figure 1). This paper reviews the contemporary knowledge of the effects of eight trace elements on OA, and explores their mechanisms in OA (Table 1).

**Table 1:** Summary table of the effects of boron, cadmium, copper, iron, magnesium, manganese, selenium, and zinc on osteoarthritis.

**Element**	**Function**	**Mechanism**	**References**
Boron	Supplementation: Preventing OA; Alleviating pain and discomfort; Reducing inflammation; Improve joint function; Repairing osteochondral defects; Deprivation: Decreasing chondrocyte density	Downregulating some enzyme activities; Inhibiting inflammatory response, reducing inflammatory cytokines; Enhancing antioxidant defense; Improving absorption of calcium, phosphorus and magnesium, and retention of calcium and magnesium	(21, 22, 26–35)
Cadmium	Increase risk of OA; Inducing cartilage degradation, resulting in osteophyte formation, subchondral sclerosis, synovitis	Reducing proteoglycan and glycosaminoglycan, inhibiting aggrecan and COL II; Producing IL-1β and IL-6, activating MMPs, destroying ECM; Aggravating homeostasis imbalance; Promoting production of ROS; Displacing essential elements, such as zinc, iron, copper, manganese, nickel, chromium	(36–43)
Copper	Relieving OA symptom; Promoting cartilage injury healing; Enhancing cartilage formation; Promoting articular cartilage and subchondral bone regeneration;	Activating cartilage immune response; Enhancing anti-inflammatory cytokine secretion, inhibiting inflammatory response, reducing cartilage tissue lesion; Restraining NO release, enhancing anti-catabolism; Improving collagen cross-linking, enhancing COL II synthesis; Increasing chondrocyte viability; Promoting chondrocyte proliferation and maturation; Raising chondrogenic gene expression, enhancing chondrogenesis differentiation of MSCs	(44–51)
	Deficiency: Destroying cartilage integrity; Increasing incidence of OA; Excess: Developing early-onset OA	Impairing cross-linking between collagen and elastin, weakening cartilage	(44, 52–54)
		Raising oxidation capacity, damaging joint	(55, 56)
Iron	Excess: Impairing cartilage; Exacerbating pathogenesis and progression of OA	Releasing pro-inflammatory cytokines, triggering chondrocyte catabolism; Inducing hydroxyl radical driven chondrocyte apoptosis and matrix decomposition; Producing ROS, causing oxidative stress, activating MMPs, degrading ECM, reducing cartilage resistance; Reducing expression of transcripts for COL II and aggrecan, increasing transcriptional counts of pro-inflammatory cytokines, establishing OA-related phenotype	(57–70)
Magnesium	Relieving mechanical allodynia and thermal hyperalgesia; Alleviating pain; Reducing cartilage degeneration and articular cartilage lesion; Retarding OA progression;	Inhibiting inflammatory cytokines and neurotransmitters; Reducing chondrocyte apoptosis; Increasing synthesis of cartilage matrix, alleviating degeneration of cartilage matrix, inducing assembly of cartilage matrix, inhibiting expression of inflammatory cytokines and proteases, promoting adhesion of synovial MSCs and collagen, raising adhesion of human synovial MSCs to osteochondral defects; Enhancing formation of chondrocytes from synovial MSCs, promoting chondrocyte proliferation and improving effect of growth factor;	(71–80)
	Deficiency: Exacerbating progression of OA; Delaying cartilage and bone differentiation; Causing lack of orderly arrangement of chondrocyte columns, reducing bone formation	Elevating CRP; Increasing proinflammatory cytokines; Activating of immune response cells; Inducing fibroblasts senescence	(81–85)
Manganese	Relieving OA symptoms, improving imaging indicators; Retarding articular cartilage degeneration, Repairing cartilage; Alleviating OA severity	Enhancing synthesis of glycosaminoglycan, proteoglycan, and COL II; Regulating metabolism of cartilage matrix; Scavenging oxygen free radical and antioxidant, reducing oxidative stress; Enhancing chondrocyte viability, protecting ECM	(86–89)
	Deficiency: Causing cartilage dysplasia	Impairing glycosaminoglycan biosynthesis	(90)
Selenium	Enhancing cartilage regeneration, improving repair of metaphyseal injury	Scavenging ROS, improving immune system function, enhancing antioxidant defense, maintaining cartilage homeostasis and redox balance; Blocking expression of pro-inflammatory gene, reducing inflammatory response; Enhancing proliferation and differentiation of chondrogenic progenitor cells; Promoting proliferation of ATDC5 chondrogenic cells	(71–78, 83–85, 91, 92)
	Deficiency: Causing joint abnormality; Increasing risk for OA	Compromising cartilage metabolism; Inducing articular fibrocartilage formation, causing cartilage degradation; Inhibiting expression of chondrogenic genes SOX9, aggrecan, and COL II, reducing the alkaline phosphatase activity, disturbing chondrogenic differentiation of ATDC5 cells; Influencing chondrogenic differentiation of MSCs, affecting endochondral ossification; Impairing homeostasis of cartilage matrix	(78–80, 93, 94)
Zinc	Alleviating OA symptom; Preventing OA progression	Reducing expression of proinflammatory cytokines, possessing anti-inflammatory activity; Scavenging ROS, exerting antioxidant effect, alleviating oxidative injury; Activating Phosphoinositide 3-kinase (PI3K)-Akt signaling pathway, increasing matrix synthesis, raising chondrocyte survival; Promoting expression of IL-10 mRNA, blocking expression of IL-1β mRNA and MMPs-13 protein, raising IL-10 levels, reducing MMPs-13 and IL-1β; Increasing chondrocyte proliferation, promoting differentiation of MSCs into chondrocytes, enhancing growth and maturation of cartilage;	(87–90, 95–97)
	Deficiency: Impairing cartilage maturation Excess: Damaging articular cartilage	Inhibiting multiplication of chondrocytes, resulting in chondrocytes disorganization;	(95)
		Up-regulating transcription of IL-6 and IL-11, inducing expression of MMPs, releasing cytokines and MMPs, causing articular cartilage injury and synovium; Activating ZIP8-Zn-MTF1 axis, increasing MMPs-13 synthesis, aggravating cartilage damage	(98–102)

*OA, osteoarthritis; COL II, type II collagen; IL-1β, interleukin-1β; IL-6, interleukin-6; IL-11, interleukin-11; ECM, extracellular matrix; MMPs, matrix metalloproteinases; ROS, reactive oxygen species; NO, nitric oxide; MSCs, mesenchymal stem cells; MTF1, metal regulatory transcription factor-1*.

## Impact of Eight Trace Elements on OA

### Boron

Dietary boron supplementation may affect the metabolism of substances such as calcium, magnesium, reactive oxygen species and reactive nitrogen (27, 28). Boron-deficient diets have been linked to OA. Korkmaz et al. (29) have indicated that in areas where boron intakes usually are 1.0 mg or less/day, incidence of OA ranges from 20 to 70%, while the incidence of OA is between 0% and 10% in areas where boron intake is usually between 3 and 10 mg. Serum boron concentration was significantly lower in patients with OA, and negatively correlated with the duration and severity of the disorder (29). Newnham et al. (30) found lower boron contents in the femur heads and synovial fluid from patients with OA as compared to those without this disease.

Oral or intraperitoneal injection of boron is beneficial to rats with induced OA (30). Compared with the placebo group, the boron supplementation trial group obtained significant favorable effects to 6 mg boron/day supplement (30). Pietrzkowski et al. (31) have demonstrated that both twice-daily 108 mg dose of calcium fructoborate group and the 216 mg calcium fructoborate in a single dose group exhibited significant improvement in the symptoms of knee OA, while no significant change was found in the placebo group. The intraarticular injection of boron significantly improved the function of patients suffering from OA (21). Boron can effectively treat OA by relieving pain and discomfort and reducing inflammation (32).

Boron can adjust inflammatory reactions in OA states by downregulating some enzyme activities at the inflammatory sites, inhibiting the inflammatory response, and influencing the production of inflammatory cytokines resulting from cartilage cells and cells involved in the inflammatory reaction (33, 34). C-reactive protein levels are related to OA progression (35). Boron, in combination with plant-derived calcium, such as calcium fructoborate, has been indicated to significantly reduce blood level of C-reactive protein in patients suffering from OA (103). Moreover, boron can inhibit simultaneously both prostaglandins and leukotrienes that are mediators in inflammatory state (104). On the other hand, some boron-compounds, such as calcium fructoborate, boric acid or borax, show promising antioxidant properties. Supplementation of boric acid or borate can improve antioxidant defense mechanism, stimulating activities of superoxide dismutase, glucose-6-phosphate dehydrogenase, catalase, glutathione peroxidase and glutathione-S-transferase enzymes, detoxifying reactive oxygen species and reactive nitrogen, reducing oxidative stress, and lowering DNA damage and lipid peroxidation (36). Furthermore, it has been found that the addition of calcium fructoborate to human diets has beneficial effects on various anti-inflammatory processes. calcium fructoborate regulates macrophage to produce inflammatory mediators, inhibits the development of endotoxin related diseases and suppresses cytokine formation. The anti-inflammatory activity of calcium fructoborate might be owing to the regulation of serine proteases released by inflammation-activated leukocytes: reducing leukotriene synthesis, lowering reactive oxygen species concentration and inhibiting the biosynthesis of arachidonic acid derived pro-inflammatory prostaglandins. Moreover, calcium fructoborate is an effective superoxide ion scavenger with anti-inflammatory activity (33, 37).

In addition, boron is helpful to prevent OA, possibly by enhancing absorption of calcium, phosphorus, and magnesium, and retention of magnesium and calcium (38). Boron can effectively repair osteochondral defects, which may make boron a promising method for the treatment of cartilage injury in OA (21).

### Cadmium

Bone is the main target organ of cadmium accumulation owing to chronic exposure (39). Krachler et al. (40) have indicated that cadmium concentration in serum of patients with OA is 0.114 mg/kg, which exceeds the maximum reference limit without occupational exposure. Cadmium can reduce the content of proteoglycan and glycosaminoglycan and inhibit the expression of type II collagen and aggrecan in the extracellular matrix of articular cartilage (41).

Bodo et al. (42) have observed that the gene expression of matrix metalloproteinase-13, which destroys extracellular matrix, increases after being exposed to a CdCl2 concentration of 1 μM for 3 days. Cadmium can aggravate the homeostasis imbalance in the joint tissue, inducing the cartilage degradation, resulting in osteophyte formation, subchondral sclerosis, chondrocyte apoptosis and synovitis (43).

Martínez-Nava et al. (23) have noted that cadmium exposure may influence negatively the level of essential elements, such as zinc, iron, manganese, nickel, chromium at the cartilage, possibly exacerbating cartilage degeneration due to the reduction of proteoglycan and glycosaminoglycan, which are key components in the extracellular matrix of articular cartilage. Epidemiological studies have showed that exposure to cadmium, may stimulate OA progression due to its ability to trigger oxidative stress (44, 105). This is because cadmium easily displaces elements such as Fe^2+^ and Cu^2+^ in the membrane and cytosolic proteins and interacts with the sulfhydryl groups in the mitochondrial membrane proteins and antioxidants, such as catalase, glutathione and superoxide dismutase, thereby promoting the production of reactive oxygen species such as hydrogen peroxide and hydroxyl radicals (41, 45). This results in production of interleukin-1β and interleukin-6, which activate matrix metalloproteinases (MMP-1, MMP-3, MMP-9, MMP-13) that destroy the extracellular matrix and affect the expression of aggrecan and type II collagen (46).

Understanding the mechanisms of cadmium on OA are of great significance for taking prevention measurements when dealing with this metal, preventing consumption of contaminated food and supporting consumption of food rich in antioxidants or essential elements, such as zinc, selenium, iron, which can counteract the harmful effects of cadmium. At present, it is only known that the level of blood cadmium is related to the duration and severity of OA, and the relationship between cadmium and OA has not been widely studied. Hence, the role of cadmium in OA should be further revealed, thereby making a direct impact conducive to preventing and avoiding the pathogenesis and progression of OA.

### Copper

Copper is essential for the normal growth, development and bone health and affects the homeostasis of cellular and humoral immunity. It is an intermediate part of the function of many immune cells and maintains the stability of the body's defense system (47). Copper can promote the regeneration of both articular cartilage and subchondral bone by activating the immune response of cartilage, which is conducive to the reconstruction of osteochondral interface and the recovery of cartilage lesion (24). The underlying mechanism may be associated with the activation of hypoxia inducible factor (HIF) mediated by copper, then further increasing the transformation of macrophages into macrophage 2 phenotype which enhances the secretion of anti-inflammatory cytokines. Consequently, copper can inhibit the inflammatory response, reduce the damage of cartilage tissue and promote the proliferation and maturation of chondrocytes (48–50). Yassin et al. (51) have demonstrated that the remission degree of OA symptoms in the copper combined with indomethacin group was better than that in the indomethacin group alone, in terms of safety and efficacy parameters, the lowest dose of copper combined with indomethacin synthetic agent is better. The experimental results verify the effective anti-inflammatory role of copper in OA.

Copper functions as a cofactor of some enzymes in cartilage, such as superoxide dismutase, cytochrome C, ascorbate oxidase, and lysyl oxidase. Particularly, lysyl oxidase, a copper-dependent amine oxidase, is a key enzyme for collagen and elastin cross-linking, which further enhances the cartilage formation (24, 106). Furthermore, Amarilio et al. (48) have noted that copper may regulate cartilage homeostasis by modulating activity of hypoxia inducible transcription factor and chondrogenic associated proteins, which is vital for chondrocyte survival. At the same time, copper reduces bone turnover by inhibiting the function of osteoblasts and osteoclasts, which is essential for the maturation of collagen tissue and is conducive to the balance and stability of joint tissue (52). In addition, copper can significantly enhance chondrogenesis differentiation of mesenchymal stem cells. Specifically, copper could promote the cytoskeleton change of mesenchymal stem cells, enhance glycosaminoglycan deposition, and significantly raise the chondrogenic gene expression including Sox9, aggrecan and collagen, resulting in considerable benefits for the application and development of cartilage repair (53).

It should be noted that copper is vital to maintain the integrity and homeostasis of cartilage tissue. The disorder of copper metabolism is closely related to the pathogenesis of OA (54). Copper deficiency results in the disorder of lysine oxidase, which impairs the cross-linking between collagen and elastin, reduces the strength of bone matrix, weakens cartilage, brings about cartilage susceptible to undergo subsequent fragmentation and damages the integrity of cartilage (25, 107, 108). Consequently, copper deficiency could reduce bone strength, impair cartilage integrity and increase the incidence of OA (24). Dietary copper supplementation has been reported to reduce the severity of osteochondrosis and other developmental cartilage lesion, possibly due to improved collagen cross-linking and enhanced synthesis of type II collagen (55). The protective effect of copper on cartilage may be attributable to the anti-catabolism of Cu^2+^ that eliminates the decomposition of cartilage matrix proteoglycan by restraining the release of nitric oxide (56). However, it has been found that patients with Wilson's disease, a hereditary disorder of copper metabolism, develop early-onset OA. This early-onset OA correlates closely with the excessive accumulation of copper in Wilson's disease (26). With the increase of free copper ion content, the oxidation capacity of copper exceeds its own antioxidant capacity, thereby damaging joints (109).

Therefore, the acquired diseases, deficiency or excess of copper in diet result in the change of copper level in human body beyond their own compensation ability, which can also cause certain damage to the joint. Consequently, the effect of copper on OA may be bidirectional. Balanced copper intake undoubtedly exerts a positive effect on OA.

### Iron

Iron is a ubiquitous element that participates in many physiological processes. It can promote collagen synthesis and the conversion of 25 hydroxyvitamin D to its active form (110, 111). Meanwhile, it stimulates the synthesis of bone matrix by activating lysyl hydroxylase, activates 25 hydroxycholecalciferol hydroxylase and supports the mineralization of bone matrix through vitamin D. Díaz-Castro et al. (57) have found that severe iron deficiency results in poor bone mineralization, alters bone microstructure, reduces bone strength, and decreases biochemical markers of bone formation, such as N-terminal peptide of type I procollagen.

However, the beneficial iron concentration window is narrow. Nugzar et al. (58) have noticed that elevated serum ferritin correlates with more severe knee cartilage injury in patients with primary OA. Higher ferritin is associated with narrower joint space (59). Yazar et al. (54) have observed that the iron concentration in synovial fluid of patients with OA is significantly higher than that of patients with rheumatoid arthritis and age-matched healthy subjects. Hereditary hemochromatosis is an iron overload disease in the body. OA is one of the most common complications in patients with hereditary hemochromatosis. Iron overload exacerbates the pathogenesis and progression of OA (60, 61). It is worth noting that osteophytes usually occur in patients with systemic iron overload caused by hemochromatosis (62). The link between osteophyte formation and iron overload may be located in the BMP/SMAD signal pathways shared by transforming growth factor 1 (TGF-1) and iron regulator hepcidin (63). Burton et al. (64) have indicated that systemic iron overload lead to iron accumulation in chondrocytes. Excessive iron is related to the early onset and progression of OA in 12-week-old Strain 13 guinea pigs. These evidences suggest that iron may play a role in the pathogenesis of OA and have a potential mechanical connection between these coinciding conditions.

The role of iron in the pathogenesis of OA has been explored in some studies. Nieuwenhuizen et al. (65) have shown that iron overload synovial cells release pro-inflammatory cytokines, such as interleukin-1, interleukin-6, and tumor necrosis factor-α, which trigger the catabolism of chondrocytes. In addition, high level of iron has been found in the infrapatellar fat pads of iron overload animals, which is owing to the accumulation in macrophages (111). The infrapatellar fat pad plays an important role in the homeostasis of knee joint (66). It is composed of adipocytes, fibroblasts and leukocytes, which is prone to become a source of inflammatory mediators that may lead to OA (67). Moreover, iron directly affects cartilage by inducing hydroxyl radical driven chondrocyte apoptosis and matrix decomposition (65, 68). Excessive iron also can produce reactive oxygen species through Fenton or Haber Weiss reaction, resulting in oxidative stress (69). This will over activate matrix metalloproteinases that degrade extracellular matrix, thereby reducing the resistance of cartilage to mechanical stress and exacerbating OA (70).

Chondrocyte exposure to iron raised the expression of matrix degrading enzymes, resulting in decreased synthesis of extracellular matrix. Cartilage degeneration brought with reduced expression of transcripts for aggrecan and type II collagen in iron overload animals (112). The cartilage injury caused by systemic iron overload is also related to the increased transcriptional counts of pro-inflammatory cytokines such as interleukin-1β and tumor necrosis factor-α in the cartilage and infrapatellar fat pad (70). Iron overload can destroy the metabolism of chondrocytes and promote the establishment of an OA-related phenotype (112). These changes of gene expression support the increase of OA related lesions in iron overload animals.

However, with the increase of age, due to the lack of the main mechanism of iron excretion in human body and the lack of effective blood circulation in articular cartilage, iron continues to accumulate in cartilage. We can speculate that the degenerative cartilage changes in middle-aged and elderly OA patients may be related to the cartilage damage caused by iron overload in the joint. Because of the complexity of the molecular mechanism of iron metabolism, there is still a relative lack of research on the relevant mechanism and signal molecules of bone and cartilage injury caused by iron overload. Further research is needed to clarify the exact mechanism of joint injury and provide new ideas for developing new strategies to prevent or reverse iron overload related cartilage damage.

### Magnesium

Magnesium is a key cofactor in any reaction driven by adenosine triphosphate (ATP) and acts as a calcium channel antagonist (113). Moreover, magnesium may be a pain mediator by altering the levels of inflammatory cytokines and neurotransmitters in the organism (81). Low-grade systemic inflammation is closely associated with the pathogenesis of OA. Konstari et al. (82) have indicated that low dietary magnesium intake is related to elevated serum C-reactive protein, which is the most sensitive biomarker of inflammation. Some proinflammatory cytokines, such as interleukin-6, tumor necrosis factor-α, increase under the condition of magnesium deficiency. The activation of immune response cells (macrophages, neutrophils, endothelial cells) is also related to magnesium deficiency. Low magnesium may be a contributing factor leading to the progression of OA via inflammatory and immune mechanisms (83, 84). Additionally, Zeng et al. (84) have demonstrated that low magnesium diet intake is related to OA. Magnesium deficiency can lead to delayed cartilage and bone differentiation (85). Hypomagnesemia has been proved to result in the lack of orderly arrangement of chondrocyte columns and reduce bone formation (91).

Ryu et al. (92) have shown that interleukin-6 is an important cytokine in the pathogenesis of OA. Latourte et al. (71) found that the blockade of interleukin-6 signaling pathway could alleviate OA in mice. Magnesium can significantly inhibit the expression of interleukin-6 in both the cartilage and synovium (72). The serum magnesium level in patients with severe OA was significantly lower than patients with mild OA (73). Serum magnesium concentration may be negatively correlated with knee radiographic OA (74).

A study on juvenile Wistar rats has indicated that magnesium supplementation can reduce articular cartilage lesions. The combination of vitamin E and magnesium has a better protective effect on articular cartilage (75). The degree of cartilage degeneration was significantly lower in OA rats injected with magnesium sulfate than that in OA rats treated with intramuscular normal saline. In OA rats treated with magnesium sulfate, thermal hyperalgesia and mechanical allodynia were relieved, and the chondrocyte apoptosis in OA rats was reduced (22). Shmagel et al. (113) have noticed that magnesium nutritional supplementation could slow down the progression of OA and relieve the pain of affected joints. Intra articular injection of magnesium can alleviate pain and reduce cartilage damage in patients with OA (76).

Magnesium can enhance the formation of chondrocytes from synovial mesenchymal stem cells, promote chondrocyte proliferation and improve the effect of growth factor through continuous effects in the process of cartilage formation (77). Baker et al. (78) have shown that the chondrocytes treated with magnesium sulfate combined with local anesthetic were more viable than those treated with local anesthetic alone. The proliferation and redifferentiation of chondrocytes increased in a dose-dependent manner after the addition of magnesium sulfate. However, with increasing extracellular magnesium concentration, cartilage formation was inhibited (77). Magnesium promoted the adhesion of synovial mesenchymal stem cells and collagen on the first day after administration, and induced the assembly of cartilage matrix 2 weeks after treatment. Moreover, magnesium increased the cartilage matrix synthesis through integrin 1 during the chondrogenesis of synovial mesenchymal stem cells *in vitro* (79). Yao et al. (72) have noted that magnesium can significantly alleviate the degeneration of cartilage matrix *in vivo* and inhibit the expression of inflammatory cytokines and proteases in articular cartilage, which may contribute to the reduction of cartilage degeneration in OA. Additionally, magnesium raises the adhesion of human synovial mesenchymal stem cells to osteochondral defects (79). Magnesium can also influence fibroblasts, more specifically, magnesium deficiency can induce cellular senescence of human fibroblasts (80).

High concentration of extracellular magnesium may suppress the mineralization of mesenchymal stem cells during osteogenic differentiation by magnesium transporter SLC41A1 (93). Magnesium can also promote the proliferation of mesenchymal stem cells and induce osteogenic differentiation by activating Notch1 signal (94). After incubation with high concentration of magnesium, the adhesion of the mesenchymal stem cells to the substrate was enhanced by phosphorylated focal adhesion kinase within the first 24 h. Then, within the next 1–2 weeks, mesenchymal stem cells were programmed to differentiate into cartilage and osteogenesis (77).

The increasing studies have demonstrated that magnesium is a promising treatment for OA. Intra articular injection of magnesium sulfate is an effective method for the treatment of OA (22, 114). However, there are not enough clinical trials to determine the potential therapeutic mechanism responsible. It is necessary to prove the clear mechanism of magnesium in OA and verify the specific method of administration *in vivo*. An in-depth understanding of the mechanism of magnesium in the treatment of OA will provide new discoveries for the self-healing process of cartilage, and design a feasible method to cure early OA.

### Manganese

Manganese is a cofactor for numerous enzymes, including mitochondrial superoxide dismutase, phosphoglucomutase and glycosyltransferase. Glycosyltransferase is involved in the synthesis of glycosaminoglycan, proteoglycan and type II collagen in cartilage extracellular matrix (86). There are high manganese concentrations that range from 1.37 μg/g to 2.21 μg/g in articular cartilage, which is necessary for the normal metabolism of articular cartilage (87).

Manganese can slow down the degeneration of articular cartilage and is beneficial to the repair of articular cartilage. Das et al. (88) have reported that the combined use of manganese, glucosamine and chondroitin can regulate the metabolism of articular cartilage matrix, relieve the symptoms of OA and improve imaging indicators. Manganese deficiency impairs glycosaminoglycan biosynthesis, resulting in cartilage dysplasia characterized by OA and deformity (90).

Oxidative stress has been implicated in the onset and progression of OA. Manganese dioxide has strong antioxidant capacity by scavenging oxygen free radical, which can reduce the oxidative stress of articular cartilage caused by inflammation and alleviate OA severity without the adverse effects of chronic use of pain medication. Moreover, Manganese dioxide can enhance chondrocyte viability and protect extracellular matrix (89). Thus, manganese dioxide can serve as a promising approach for cartilage protection in OA.

### Selenium

Selenium supplement has become an indispensable part in the process of health care for many people (95). Significant health benefits have been attributed to selenium which exerts important role in the thyroid hormone metabolism, male reproduction, immune function, anti-inflammation and free radical detoxification (96–100). However, selenium deficiency is related to impaired immune system and increased susceptibility to various diseases such as OA (101, 102, 115). Some studies have indicated detrimental effects of selenium deficiency, such as growth retardation, compromised bone and cartilage metabolism, joint abnormalities (116, 117). Mice fed a selenium deficient diet induced articular fibrocartilage formation, eventually resulting in articular cartilage degradation (118). Selenium level in the serum of patients with OA is significantly lower than that of normal controls (119). Likewise, Jordan et al. (120) have showed the link between low selenium contents in toenails and OA related pain and disease severity. It is apparent that selenium deficiency may be a potential risk factor for OA.

Several studies have shown that oxidative load accumulates in chondrocytes undergoing changes in OA (120, 121). The harmful effects of reactive oxygen species on cartilage homeostasis can be effectively relieved by enhancing cellular antioxidant activity, and OA can be treated through targeting the regulatory factors involved in cartilage oxidative stress (122, 123). The abnormal production of reactive oxygen species and the impairment of cellular antioxidant capacity result in oxidative stress. Selenium is incorporated into selenoprotein for forming the active site of selenoenzymes. One of selenoenzymes is glutathione peroxidase, which is involved in scavenging reactive oxygen species (124). Meanwhile, selenium can protect chondrocytes from oxidative injury. The protective effect of selenium on cartilage is mainly attributed to antioxidant defense function (125). Additionally, selenium appears in the form of selenomethionine, which can block the expression of pro-inflammatory gene induced by interleukin-1β, thereby reducing the inflammatory response (126).

Selenium plays beneficial roles on enhancing proliferation and differentiation of chondrogenic progenitor cells (127). The ATDC5 cell line is originated from mouse teratoma cells and characterized by a chondrogenic cell line, which experiences a sequential process similar to chondrocyte differentiation. ATDC5 chondrocytes are differentiated from ATDC5 cell line (128). Selenium supplementation promoted the proliferation of ATDC5 chondrogenic cells by inducing the expression of cyclin D1, even under serum deprivation (129). Selenium deficiency disturbed the chondrogenic differentiation of ATDC5 cells by inhibiting the expression of chondrogenic genes SOX9, aggrecan, and type II collagen and reducing the alkaline phosphatase activity (130). Consistent with the impacts of selenium and selenoprotein on chondrogenic progenitor cells observed *in vitro*, deficient intake of selenium seriously influenced chondrogenic differentiation of mesenchymal stem cells, thereby affecting the endochondral ossification of mice (116). Sun et al. (131) have shown that selenium deficiency impairs the homeostasis of cartilage matrix. Low selenium decreased the expression level of type II collagen regulated by SOX9, which is known as the main regulator required for keeping cartilage matrix homeostasis. Selenium deficiency stimulated the expression of the chondrocyte hypertrophy marker gene type X collagen in the articular cartilage (116). The expression of parathyroid hormone related protein (PTHrP), which inhibits chondrocyte maturation in the period of endochondral ossification, increased in the hypertrophic growth plate and articular cartilage following selenium deficiency (132).

Selenium can improve the antioxidant activity of chondrocytes under oxidative stress, scavenging reactive oxygen species, reducing the adverse effect of reactive oxygen species on cartilage homeostasis, relieving the cartilage damage induced by oxidative stress, thereby protecting cartilage. Moreover, selenium can enhance cartilage regeneration, improve the repair of metaphyseal injury, and increase the proliferation and differentiation of cartilage progenitor cells. In addition, selenium can improve immune system function, enhance antioxidant defense, and maintain cartilage homeostasis and redox balance. Therefore, strategies aimed at optimizing the role of selenium should be considered in the prevention and treatment of OA, and selenium supplementation plans should be carefully formulated to enhance the benefit of selenium in OA.

### Zinc

Zinc is a component of more than 300 enzymes and participates in synthesis of protein, DNA and RNA by zinc finger proteins (133, 134). Zinc is also involved in oxidative stress, homeostasis, immune responses, aging and apoptosis (135, 136). Moreover, zinc is a co-factor of superoxide dismutase (SOD), an inducer of metallothionein, and an inhibitor of nicotinamide adenine dinucleotide phosphate (NADPH) oxidase. It is essential for antioxidant defense and suppressing prostaglandin synthesis (137, 138). Moreover, zinc, which is known as zinc signal, is regarded as a regulator in immunity and redox metabolic signaling pathways (139). Zinc enhances the growth and maturation of cartilage, improving the activity of vitamin D and stimulating synthesis of metallothionein (85). Zinc has additional benefits in promoting the differentiation of mesenchymal stem cells into chondrocytes in osteochondral defects (140). Rodríguez and Rosselot (141) have demonstrated that low dose of zinc (<0.5 μM) can increase the cultured chondrocyte proliferation by 40–50%. While dietary zinc deficiency inhibits the multiplication of chondrocytes and leads to chondrocyte disorganization, which may be associated with matrix metalloproteinase disorder (142).

Glutathione is the first line of defense against oxidative injury and can scavenge reactive oxygen species. The reactive oxygen species catalyze the production of hydrogen peroxide from oxygen anions, which is subsequently decomposed into water by glutathione peroxidase or catalase. All of these are called the intracellular antioxidant defense systems (143). Kloubert et al. (144) have noticed that zinc is necessary for the activation of these defense enzymes. Zinc exerts anti-inflammatory effect through reducing the expression of proinflammatory cytokines, which can alleviate OA (145). Moreover, zinc supplementation significantly promotes the expression of interleukin-10 mRNA and blocks the expression of interleukin-1β mRNA and matrix metalloproteinases-13 protein, thereby massively raising serum interleukin-10 levels and reducing matrix metalloproteinases-13 and interleukin-1β levels (146). Interleukin-10 disturbs the secretion of proinflammatory cytokines and decreases the production of matrix metalloproteinases in chondrocytes (147). Additionally, nuclear factor erythroid 2-related factor (Nrf2) is a main regulator of antioxidant defense genes that can adjust the redox disorder by the upregulation of responsive antioxidant enzymes (148). The activation of the Phosphoinositide 3-kinase(PI3K)-Akt signaling pathway can increase matrix synthesis and raise chondrocyte survival (149). Zinc may enhance nuclear factor erythroid 2-related factor(Nrf2) expression and promote the upstream active regulator, Phosphorylated-Akt (p-Akt) expression, showing that the Phosphoinositide 3-kinase(PI3K)-Akt/nuclear factor erythroid 2-related factor(Nrf2) pathway is associated with the zinc actions (150). Huang et al. (133) revealed that zinc supplementation of 1.6 mg/kg/day was sufficient to prevent the progression of OA, while zinc supplementation of 8.0 mg/kg/day had no better effect.

However, zinc overload damages articular cartilage, for example, increased levels of zinc ion, ZIP8 protein and its related mRNA are common in OA articular cartilage. zinc participates in zinc finger protein Zac1 up-regulating the transcription of interleukin-1β through interleukin-1 family proteins, which directly induces the expression of matrix metalloproteinase. The release of cytokines and matrix metalloproteinases causes the injury of articular cartilage and synovium (151, 152). Moreover, when the ZIP8-Zn-MTF1 (metal regulatory transcription factor-1) axis is activated, the increased synthesis of matrix metalloproteinase-13 will aggravate the cartilage damage of OA (151, 153). In addition, the overexpression of ZIP8 results in the increase of zinc influx in chondrocytes, which enhances the activity of zinc dependent matrix metalloproteinases, inducing cartilage rupture. The cartilage fragments resulting from these enzymes are subsequently recognized as danger signals by immune cells, which triggers intimal inflammation, known as synovitis, which feedbacks to further exacerbate the response (154).

## Conclusions

Osteoarthritis (OA) is a degenerative joint disease, with an age-associated increase in both incidence and prevalence. The hallmark of OA is pathological changes of the joint structure, such as cartilage erosion and synovial inflammation. So far, no efficient treatment can alter the pathological progression of OA. The current therapy roughly include pharmaceutical and non-pharmaceutical approaches prior to surgical intervention. However, its morbidity cannot be reduced using these measures, suggesting non- pharmaceutical prevention may be achieved by lifestyle and nutrition. As described in this article, OA is associated with trace elements in the environment, which can be essential, deleterious, or both, depending on the type and level of trace elements (Table 1). Undoubtedly, many trace elements affect the onset and progression of OA. Some trace elements such as boron and magnesium are conducive to the prevention and treatment of OA. By contrast, other trace elements like cadmium are toxic even at low concentrations and can induce OA. Furthermore, it is worth noting that some trace elements are dose-dependent, and the safe concentration range of these trace elements is narrow. Deficiency or excess of trace elements such as copper may bring about OA (Figure 1). To a certain extent, the status of trace elements depends on the external environment (nutrition) and internal factors (individual absorption and metabolism of trace elements, genetic tendency, age and gender). The roles of trace elements depend not only on their content, but also on the interaction among them. Though recent advances in knowledge in this area, much remains to be discovered to fully elucidate the effects of trace elements on OA. The protective effects of selected trace elements on OA have been found. However, theoretical and practical issues of supplementation in the prevention and treatment of OA are still unknown. Data concerning the impact of individual trace element is limited, partly due to the obtainment of these elements in combination. Additionally, the homeostasis of trace elements in organisms depends on various factors such as diet, age and gender. Further studies are required for confirming the effects of different doses, concentrations and interactions of individual trace element on OA, thereby, preventing and treating OA.

## Author Contributions

All authors have contributed to the design, interpretation of data, writing, and supervision of the final version of this manuscript.

## Conflict of Interest

The authors declare that the research was conducted in the absence of any commercial or financial relationships that could be construed as a potential conflict of interest.

## Publisher's Note

All claims expressed in this article are solely those of the authors and do not necessarily represent those of their affiliated organizations, or those of the publisher, the editors and the reviewers. Any product that may be evaluated in this article, or claim that may be made by its manufacturer, is not guaranteed or endorsed by the publisher.

## References

[1] Carlson AK Rawle RA Wallace CW Brooks EG Adams E Greenwood MC. Characterization of synovial fluid metabolomic phenotypes of cartilage morphological changes associated with osteoarthritis. Osteoarthritis Cartilage. (2019) 27:1174–84. 10.1016/j.joca.2019.04.007 31028882 PMC6646055

[2] Kosik-Bogacka DI Lanocha-Arendarczyk N Kot K Ciosek Z Zietek P Karaczun M. Effects of biological factors and health condition on mercury and selenium concentrations in the cartilage, meniscus and anterior cruciate ligament. J Trace Elem Med Biol. (2017) 44:201–8. 10.1016/j.jtemb.2017.08.008 28965577

[3] GBD 2017 Disease and Injury Incidence and Prevalence Collaborators. Global, regional, and national incidence, prevalence, and years lived with disability for 354 diseases and injuries for 195 countries and territories, 1990-2017: a systematic analysis for the Global Burden of Disease Study 2017. Lancet (London, England). (2018) 392:1789–858. 10.1016/S0140-6736(18)32279-7 30496104 PMC6227754

[4] Misra D Fielding RA Felson DT Niu J Brown C Nevitt M. Risk of knee osteoarthritis with obesity, sarcopenic obesity, and sarcopenia. Arthritis Rheumatol (Hoboken, NJ). (2019) 71:232–7. 10.1002/art.40692 30106249 PMC6374038

[5] Al-Mahrouqi MM Vicenzino B MacDonald DA Smith MD. Disability, physical impairments, and poor quality of life, rather than radiographic changes, are related to symptoms in individuals with ankle osteoarthritis: a cross-sectional laboratory study. J Orthop Sports Phys Ther. (2020) 50:711–22. 10.2519/jospt.2020.9376 33256512

[6] Primorac D Molnar V Rod E Jeleč Ž Cukelj F Matišić V. Knee osteoarthritis: a review of pathogenesis and state-of-the-art non-operative therapeutic considerations. Genes. (2020) 11:854. 10.3390/genes11080854 32722615 PMC7464436

[7] Boutefnouchet T Puranik G Holmes E Bell KM. Hylan GF-20 viscosupplementation in the treatment of symptomatic osteoarthritis of the knee: clinical effect survivorship at 5 years. Knee Surg Relat Res. (2017) 29:129–36. 10.5792/ksrr.16.061 28545178 PMC5450575

[8] Hiligsmann M Cooper C Arden N Boers M Branco JC Luisa Brandi M. Health economics in the field of osteoarthritis: an expert's consensus paper from the European Society for Clinical and Economic Aspects of Osteoporosis and Osteoarthritis (ESCEO). Semin Arthritis Rheum. (2013) 43:303–13. 10.1016/j.semarthrit.2013.07.003 23992801

[9] Henrotin Y Lambert C Richette P. Importance of synovitis in osteoarthritis: evidence for the use of glycosaminoglycans against synovial inflammation. Semin Arthritis Rheum. (2014) 43:579–87. 10.1016/j.semarthrit.2013.10.005 24262930

[10] Grenier S Bhargava MM Torzilli PA. An *in vitro* model for the pathological degradation of articular cartilage in osteoarthritis. J Biomech. (2014) 47:645–52. 10.1016/j.jbiomech.2013.11.050 24360770 PMC3938093

[11] Shepherd C Skelton AJ Rushton MD Reynard LN Loughlin J. Expression analysis of the osteoarthritis genetic susceptibility locus mapping to an intron of the MCF2L gene and marked by the polymorphism rs11842874. BMC Med Genet. (2015) 16:108. 10.1186/s12881-015-0254-2 26584642 PMC4653905

[12] Ji Q Zheng Y Zhang G Hu Y Fan X Hou Y. Single-cell RNA-seq analysis reveals the progression of human osteoarthritis. Ann Rheum Dis. (2019) 78:100–10. 10.1136/annrheumdis-2017-212863 30026257 PMC6317448

[13] Zhao L Huang J Fan Y Li J You T He S. Exploration of CRISPR/Cas9-based gene editing as therapy for osteoarthritis. Ann Rheum Dis. (2019) 78:676–82. 10.1136/annrheumdis-2018-214724 30842121 PMC6621547

[14] Rehling T Bjørkman AD Andersen MB Ekholm O Molsted S. Diabetes is associated with musculoskeletal pain, osteoarthritis, osteoporosis, and rheumatoid arthritis. J Diabetes Res. (2019) 2019:6324348. 10.1155/2019/6324348 31886282 PMC6925775

[15] Prieto-Alhambra D Judge A Javaid MK Cooper C Diez-Perez A Arden NK. Incidence and risk factors for clinically diagnosed knee, hip and hand osteoarthritis: influences of age, gender and osteoarthritis affecting other joints. Ann Rheum Dis. (2014) 73:1659–64. 10.1136/annrheumdis-2013-203355 23744977 PMC3875433

[16] Wellsandt E Zeni JA Axe MJ Snyder-Mackler L. Hip joint biomechanics in those with and without post-traumatic knee osteoarthritis after anterior cruciate ligament injury. Clin Biomech (Bristol, Avon). (2017) 50:63–9. 10.1016/j.clinbiomech.2017.10.001 28987873 PMC5718058

[17] Cerqueira MS de Brito Vieira WH. Effects of blood flow restriction exercise with very low load and low volume in patients with knee osteoarthritis: protocol for a randomized trial. Trials. (2019) 20:135. 10.1186/s13063-019-3238-2 30777115 PMC6379934

[18] Musco N Vassalotti G Mastellone V Cortese L Della Rocca G Molinari ML. Effects of a nutritional supplement in dogs affected by osteoarthritis. Vet Med Sci. (2019) 5:325–35. 10.1002/vms3.182 31313893 PMC6682793

[19] Smith MV Nepple JJ Wright RW Matava MJ Brophy RH. Knee osteoarthritis is associated with previous meniscus and anterior cruciate ligament surgery among elite college American football athletes. Sports Health. (2017) 9:247–51. 10.1177/1941738116683146 27940573 PMC5435150

[20] Zaneta C Danuta KB Natalia Ł A Karolina K Maciej K Paweł Z. Concentration of selected elements in the infrapatellar fat pad of patients with a history of total knee arthroplasty. Int J Environ Res Public Health. (2019) 16:1734. 10.3390/ijerph16040525 31100903 PMC6572265

[21] Korkmaz M Turkmen R Demirel HH Saritas ZK. Effect of boron on the repair of osteochondral defect and oxidative stress in rats: an experimental study. Biol Trace Elem Res. (2019) 187:425–33. 10.1007/s12011-018-1381-3 29869015

[22] Lee CH Wen ZH Chang YC Huang SY Tang CC Chen WF. Intra-articular magnesium sulfate (MgSO4) reduces experimental osteoarthritis and nociception: association with attenuation of N-methyl-D-aspartate (NMDA) receptor subunit 1 phosphorylation and apoptosis in rat chondrocytes. Osteoarthritis Cartilage. (2009) 17:1485–93. 10.1016/j.joca.2009.05.006 19490963

[23] Martínez-Nava GA Mendoza-Soto L Fernández-Torres J Zamudio-Cuevas Y Reyes-Hinojosa D Plata-Rodríguez R. Effect of cadmium on the concentration of essential metals in a human chondrocyte micromass culture. J Trace Elem Med. (2020) 62:126614. 10.1016/j.jtemb.2020.126614 32682287

[24] Lin R Deng C Li X Liu Y Zhang M Qin C. Copper-incorporated bioactive glass-ceramics inducing anti-inflammatory phenotype and regeneration of cartilage/bone interface. Theranostics. (2019) 9:6300–13. 10.7150/thno.36120 31534552 PMC6735521

[25] Smith BJ King JB Lucas EA Akhter MP Arjmandi BH Stoecker BJ. Skeletal unloading and dietary copper depletion are detrimental to bone quality of mature rats. J Nutr. (2002) 132:190–6. 10.1093/jn/132.2.190 11823577

[26] Ye S Dai T Leng B Tang L Jin L Cao L. Genotype and clinical course in 2 Chinese Han siblings with Wilson disease presenting with isolated disabling premature osteoarthritis: a case report. Medicine. (2017) 96:e8641. 10.1097/MD.0000000000008641 29381936 PMC5708935

[27] Sharma A Mani V Pal RP Sarkar S Datt C. Boron supplementation in peripartum Murrah buffaloes: the effect on calcium homeostasis, bone metabolism, endocrine and antioxidant status. J Trace Elem Med Biol. (2020) 62:126623. 10.1016/j.jtemb.2020.126623 32739828

[28] Acaroz U Ince S Arslan-Acaroz D Gurler Z Kucukkurt I Demirel HH. The ameliorative effects of boron against acrylamide-induced oxidative stress, inflammatory response, and metabolic changes in rats. Food Chem Toxicol. (2018) 118:745–52. 10.1016/j.fct.2018.06.029 29913234

[29] Korkmaz M Sayli U Sayli BS Bakirdere S Titretir S Yavuz Ataman O. Estimation of human daily boron exposure in a boron-rich area. Br J Nutr. (2007) 98:571–5. 10.1017/S000711450770911X 17419890

[30] Newnham RE. Essentiality of boron for healthy bones and joints. Environ Health Perspect. (1994) 102(Suppl. 7):83–5. 10.1289/ehp.94102s783 7889887 PMC1566627

[31] Pietrzkowski Z Phelan MJ Keller R Shu C Argumedo R Reyes-Izquierdo T. Short-term efficacy of calcium fructoborate on subjects with knee discomfort: a comparative, double-blind, placebo-controlled clinical study. Clin Interv Aging. (2014) 9:895–9. 10.2147/CIA.S64590 24940052 PMC4051624

[32] Li X Wang X Liu Q Yan J Pan D Wang L. ROS-responsive boronate-stabilized polyphenol-poloxamer 188 assembled dexamethasone nanodrug for macrophage repolarization in osteoarthritis treatment. Adv Healthc Mater. (2021) 10:e2100883. 10.1002/adhm.202100883 34137218

[33] Scorei RI Ciofrangeanu C Ion R Cimpean A Galateanu B Mitran V. *In vitro* effects of calcium fructoborate upon production of inflammatory mediators by LPS-stimulated RAW 264. 7 macrophages. Biol Trace Elem Res. (2010) 135:334–44. 10.1007/s12011-009-8488-5 19669712

[34] Gallardo-Williams MT Chapin RE King PE Moser GJ Goldsworthy TL Morrison JP. Boron supplementation inhibits the growth and local expression of IGF-1 in human prostate adenocarcinoma (LNCaP) tumors in nude mice. Toxicol Pathol. (2004) 32:73–8. 10.1080/01926230490260899 14713551

[35] Pearle AD Scanzello CR George S Mandl LA DiCarlo EF Peterson M. Elevated high-sensitivity C-reactive protein levels are associated with local inflammatory findings in patients with osteoarthritis. Osteoarthritis Cartilage. (2007) 15:516–23. 10.1016/j.joca.2006.10.010 17157039

[36] Wang S Yu S Feng J Liu S. A highly efficient antioxidant based on boron and a Schiff base bridged phenolic diphenylamine: synthesis, crystal structure and thermal and antioxidant properties. Acta Crystallogr Sect C Struct Chem. (2019) 75(Pt 9):1274–9. 10.1107/S2053229619011331 31484816

[37] Mogoşanu GD Bită A Bejenaru LE Bejenaru C Croitoru O Rău G. Calcium fructoborate for bone and cardiovascular health. Biol Trace Elem Res. (2016) 172:277–81. 10.1007/s12011-015-0590-2 26686846 PMC4930945

[38] Sheng MH Taper LJ Veit H Thomas EA Ritchey SJ Lau KH. Dietary boron supplementation enhances the effects of estrogen on bone mineral balance in ovariectomized rats. Biol Trace Elem Res. (2001) 81:29–45. 10.1385/BTER:81:1:29 11508330

[39] Knani L Venditti M Kechiche S Banni M Messaoudi I Minucci S. Melatonin protects bone against cadmium-induced toxicity via activation of Wnt/β-catenin signaling pathway. Toxicol Mech Methods. (2020) 30:237–45. 10.1080/15376516.2019.1701595 31809235

[40] Krachler M Domej W Irgolic KJ. Concentrations of trace elements in osteoarthritic knee-joint effusions. Biol Trace Elem Res. (2000) 75:253–63. 10.1385/BTER:75:1-3:253 11051615

[41] Yessica Eduviges ZC Martínez-Nava G Reyes-Hinojosa D Mendoza-Soto L Fernández-Torres J López-Reyes A. Impact of cadmium toxicity on cartilage loss in a 3D *in vitro* model. Environ Toxicol Pharmacol. (2020) 74:103307. 10.1016/j.etap.2019.103307 31830724

[42] Bodo M Balloni S Lumare E Bacci M Calvitti M Dell'Omo M. Effects of sub-toxic Cadmium concentrations on bone gene expression program: results of an in vitro study. Toxicol In Vitro. (2010) 24:1670–80. 10.1016/j.tiv.2010.05.020 20570719

[43] Ding C Cicuttini F Blizzard L Jones G. Smoking interacts with family history with regard to change in knee cartilage volume and cartilage defect development. Arthritis Rheum. (2007) 56:1521–8. 10.1002/art.22591 17469130

[44] Chen X Wang G Li X Gan C Zhu G Jin T. Environmental level of cadmium exposure stimulates osteoclasts formation in male rats. Food Chem Toxicol. (2013) 60:530–5. 10.1016/j.fct.2013.08.017 23954550

[45] Moulis JM. Cellular mechanisms of cadmium toxicity related to the homeostasis of essential metals. Biometals. (2010) 23:877–96. 10.1007/s10534-010-9336-y 20524046

[46] Gu J Li S Wang G Zhang X Yuan Y Liu X. Cadmium toxicity on chondrocytes and the palliative effects of 1α, 25-dihydroxy vitamin D(3) in white leghorns chicken's embryo. Front Vet Sci. (2021) 8:637369. 10.3389/fvets.2021.637369 33644155 PMC7902530

[47] Djoko KY Ong CL Walker MJ McEwan AG. The role of copper and zinc toxicity in innate immune defense against bacterial pathogens. J Biol Chem. (2015) 290:18954–61. 10.1074/jbc.R115.647099 26055706 PMC4521016

[48] Amarilio R Viukov SV Sharir A Eshkar-Oren I Johnson RS Zelzer E. HIF1alpha regulation of Sox9 is necessary to maintain differentiation of hypoxic prechondrogenic cells during early skeletogenesis. Development (Cambridge, England). (2007) 134:3917–28. 10.1242/dev.008441 17913788

[49] Robins JC Akeno N Mukherjee A Dalal RR Aronow BJ Koopman P. Hypoxia induces chondrocyte-specific gene expression in mesenchymal cells in association with transcriptional activation of Sox9. Bone. (2005) 37:313–22. 10.1016/j.bone.2005.04.040 16023419

[50] Chen X Hu JG Huang YZ Li S Li SF Wang M. Copper promotes the migration of bone marrow mesenchymal stem cells via Rnd3-dependent cytoskeleton remodeling. J Cell Physiol. (2020) 235:221–31. 10.1002/jcp.28961 31187497

[51] Yassin NZ El-Shenawy SM Abdel-Rahman RF Yakoot M Hassan M Helmy S. Effect of a topical copper indomethacin gel on inflammatory parameters in a rat model of osteoarthritis. Drug Des Devel Ther. (2015) 9:1491–8. 10.2147/DDDT.S79957 25792809 PMC4362896

[52] Roczniak W Brodziak-Dopierała B Cipora E Jakóbik-Kolon A Kluczka J Babuśka-Roczniak M. Factors that affect the content of cadmium, nickel, copper and zinc in tissues of the knee joint. Biol Trace Elem Res. (2017) 178:201–9. 10.1007/s12011-016-0927-5 28070864 PMC5506214

[53] Xu C Chen J Li L Pu X Chu X Wang X. Promotion of chondrogenic differentiation of mesenchymal stem cells by copper: implications for new cartilage repair biomaterials. Mater Sci Eng C Mater Biol Appl. (2018) 93:106–14. 10.1016/j.msec.2018.07.074 30274037

[54] Yazar M Sarban S Kocyigit A Isikan UE. Synovial fluid and plasma selenium, copper, zinc, and iron concentrations in patients with rheumatoid arthritis and osteoarthritis. Biol Trace Elem Res. (2005) 106:123–32. 10.1385/BTER:106:2:123 16116244

[55] Yuan X Wang J Zhu X Zhang Z Ai Y Sun G. Effect of copper on levels of collagen and alkaline phosphatase activity from chondrocytes in newborn piglets *in vitro*. Biol Trace Elem Res. (2011) 144:597–605. 10.1007/s12011-011-9151-5 21789542

[56] Gee E Davies M Firth E Jeffcott L Fennessy P Mogg T. Osteochondrosis and copper: histology of articular cartilage from foals out of copper supplemented and non-supplemented dams. Vet J (London, England: 1997). (2007) 173:109–17. 10.1016/j.tvjl.2005.09.015 16314126

[57] Díaz-Castro J López-Frías MR Campos MS López-Frías M Alférez MJ Nestares T. Severe nutritional iron-deficiency anaemia has a negative effect on some bone turnover biomarkers in rats. Eur J Nutr. (2012) 51:241–7. 10.1007/s00394-011-0212-5 21647667

[58] Nugzar O Zandman-Goddard G Oz H Lakstein D Feldbrin Z Shargorodsky M. The role of ferritin and adiponectin as predictors of cartilage damage assessed by arthroscopy in patients with symptomatic knee osteoarthritis. Best Pract Res Clin Rheumatol. (2018) 32:662–8. 10.1016/j.berh.2019.04.004 31203924

[59] Kennish L Attur M Oh C Krasnokutsky S Samuels J Greenberg JD. Age-dependent ferritin elevations and HFE C282Y mutation as risk factors for symptomatic knee osteoarthritis in males: a longitudinal cohort study. BMC Musculoskelet Disord. (2014) 15:8. 10.1186/1471-2474-15-8 24401005 PMC3893611

[60] Sahinbegovic E Dallos T Aigner E Axmann R Engelbrecht M Schöniger-Hekele M. Hereditary hemochromatosis as a risk factor for joint replacement surgery. Am J Med. (2010) 123:659–62. 10.1016/j.amjmed.2010.01.024 20609690

[61] Oppl B Husar-Memmer E Pfefferkorn S Blank M Zenz P Gollob E. HFE hemochromatosis screening in patients with severe hip osteoarthritis: a prospective cross-sectional study. PLoS ONE. (2018) 13:e0207415. 10.1371/journal.pone.0207415 30427934 PMC6235364

[62] Nguyen CD Morel V Pierache A Lion G Cortet B Flipo RM. Bone and joint complications in patients with hereditary hemochromatosis: a cross-sectional study of 93 patients. Ther Adv Musculoskeletal Dis. (2020) 12:1759720x20939405. 10.1177/1759720X20939405 32728396 PMC7366396

[63] Wang RH Li C Xu X Zheng Y Xiao C Zerfas P. A role of SMAD4 in iron metabolism through the positive regulation of hepcidin expression. Cell Metab. (2005) 2:399–409. 10.1016/j.cmet.2005.10.010 16330325

[64] Burton LH Radakovich LB Marolf AJ Santangelo KS. Systemic iron overload exacerbates osteoarthritis in the strain 13 guinea pig. Osteoarthritis Cartilage. (2020) 28:1265–75. 10.1016/j.joca.2020.06.005 32629162 PMC7484276

[65] Nieuwenhuizen L Schutgens RE van Asbeck BS Wenting MJ van Veghel K Roosendaal G. Identification and expression of iron regulators in human synovium: evidence for upregulation in haemophilic arthropathy compared to rheumatoid arthritis, osteoarthritis, and healthy controls. Haemophilia. (2013) 19:e218–27. 10.1111/hae.12208 23777533

[66] Wu J Kuang L Chen C Yang J Zeng WN Li T. miR-100-5p-abundant exosomes derived from infrapatellar fat pad MSCs protect articular cartilage and ameliorate gait abnormalities via inhibition of mTOR in osteoarthritis. Biomaterials. (2019) 206:87–100. 10.1016/j.biomaterials.2019.03.022 30927715

[67] Iwata M Ochi H Hara Y Tagawa M Koga D Okawa A. Initial responses of articular tissues in a murine high-fat diet-induced osteoarthritis model: pivotal role of the IPFP as a cytokine fountain. PLoS ONE. (2013) 8:e60706. 10.1371/journal.pone.0060706 23593289 PMC3625196

[68] Hooiveld MJ Roosendaal G van den Berg HM Bijlsma JW Lafeber FP. Haemoglobin-derived iron-dependent hydroxyl radical formation in blood-induced joint damage: an *in vitro* study. Rheumatology (Oxford, England). (2003) 42:784–90. 10.1093/rheumatology/keg220 12730540

[69] Yauger YJ Bermudez S Moritz KE Glaser E Stoica B Byrnes KR. Iron accentuated reactive oxygen species release by NADPH oxidase in activated microglia contributes to oxidative stress *in vitro*. J Neuroinflammation. (2019) 16:41. 10.1186/s12974-019-1430-7 30777083 PMC6378754

[70] Jing X Du T Li T Yang X Wang G Liu X. The detrimental effect of iron on OA chondrocytes: importance of pro-inflammatory cytokines induced iron influx and oxidative stress. J Cell Mol Med. (2021) 25:5671–80. 10.1111/jcmm.16581 33942503 PMC8184674

[71] Latourte A Cherifi C Maillet J Ea HK Bouaziz W Funck-Brentano T. Systemic inhibition of IL-6/Stat3 signalling protects against experimental osteoarthritis. Ann Rheum Dis. (2017) 76:748–55. 10.1136/annrheumdis-2016-209757 27789465

[72] Yao H Xu JK Zheng NY Wang JL Mok SW Lee YW. Intra-articular injection of magnesium chloride attenuates osteoarthritis progression in rats. Osteoarthritis Cartilage. (2019) 27:1811–21. 10.1016/j.joca.2019.08.007 31536815

[73] Coşkun Benlidayi I Gökçen N Sarpel T. Serum magnesium level is not associated with inflammation in patients with knee osteoarthritis. Turkish J Phys Med Rehabil. (2017) 63:249–52. 10.5606/tftrd.2017.511 31453461 PMC6648255

[74] Zeng C Wei J Li H Yang T Zhang FJ Pan D. Relationship between serum magnesium concentration and radiographic knee osteoarthritis. J Rheumatol. (2015) 42:1231–6. 10.3899/jrheum.141414 26034158

[75] Pfister K Mazur D Vormann J Stahlmann R. Diminished ciprofloxacin-induced chondrotoxicity by supplementation with magnesium and vitamin E in immature rats. Antimicrob Agents Chemother. (2007) 51:1022–7. 10.1128/AAC.01175-06 17210779 PMC1803142

[76] Kuang X Chiou J Lo K Wen C. Magnesium in joint health and osteoarthritis. Nutr Res (New York, NY). (2021) 90:24–35. 10.1016/j.nutres.2021.03.002 34023805

[77] Feyerabend F Witte F Kammal M Willumeit R. Unphysiologically high magnesium concentrations support chondrocyte proliferation and redifferentiation. Tissue Eng. (2006) 12:3545–56. 10.1089/ten.2006.12.3545 17518690

[78] Baker JF Byrne DP Walsh PM Mulhall KJ. Human chondrocyte viability after treatment with local anesthetic and/or magnesium: results from an *in vitro* study. Arthroscopy. (2011) 27:213–7. 10.1016/j.arthro.2010.06.029 20952146

[79] Shimaya M Muneta T Ichinose S Tsuji K Sekiya I. Magnesium enhances adherence and cartilage formation of synovial mesenchymal stem cells through integrins. Osteoarthritis Cartilage. (2010) 18:1300–9. 10.1016/j.joca.2010.06.005 20633668

[80] Killilea DW Ames BN. Magnesium deficiency accelerates cellular senescence in cultured human fibroblasts. Proc Natl Acad Sci U S A. (2008) 105:5768–73. 10.1073/pnas.0712401105 18391207 PMC2311331

[81] Simental-Mendía LE Rodríguez-Morán M Guerrero-Romero F. Oral magnesium supplementation decreases C-reactive protein levels in subjects with prediabetes and hypomagnesemia: a clinical randomized double-blind placebo-controlled trial. Arch Med Res. (2014) 45:325–30. 10.1016/j.arcmed.2014.04.006 24814039

[82] Konstari S Sares-Jäske L Heliövaara M Rissanen H Knekt P Arokoski J. Dietary magnesium intake, serum high sensitivity C-reactive protein and the risk of incident knee osteoarthritis leading to hospitalization-A cohort study of 4,953 Finns. PLoS ONE. (2019) 14:e0214064. 10.1371/journal.pone.0214064 30908508 PMC6433216

[83] Belluci MM de Molon RS Rossa C Jr Tetradis S Giro G Cerri PS. Severe magnesium deficiency compromises systemic bone mineral density and aggravates inflammatory bone resorption. J Nutr Biochem. (2020) 77:108301. 10.1016/j.jnutbio.2019.108301 31825817

[84] Zeng C Li H Wei J Yang T Deng ZH Yang Y. Association between dietary magnesium intake and radiographic knee osteoarthritis. PLoS ONE. (2015) 10:e0127666. 10.1371/journal.pone.0127666 26010333 PMC4444049

[85] Kosik-Bogacka DI Lanocha-Arendarczyk N Kot K Zietek P Karaczun M Prokopowicz A. Calcium, magnesium, zinc and lead concentrations in the structures forming knee joint in patients with osteoarthritis?. J Trace Elem Med Biol. (2018) 50:409–14. 10.1016/j.jtemb.2018.08.007 30262313

[86] Vásquez-Procopio J Osorio B Cortés-Martínez L Hernández-Hernández F Medina-Contreras O Ríos-Castro E. Intestinal response to dietary manganese depletion in Drosophila. Metallomics. (2020) 12:218–40. 10.1039/C9MT00218A 31799578

[87] Brodziak-Dopierała B Kwapuliński J Sobczyk K Wiechuła D. The content of manganese and iron in hip joint tissue. J Trace Elem Med Biol. (2013) 27:208–12. 10.1016/j.jtemb.2012.12.005 23415599

[88] Das A Jr. Hammad TA. Efficacy of a combination of FCHG49 glucosamine hydrochloride, TRH122 low molecular weight sodium chondroitin sulfate and manganese ascorbate in the management of knee osteoarthritis. Osteoarthritis Cartilage. (2000) 8:343–50. 10.1053/joca.1999.0308 10966840

[89] Kumar S Adjei IM Brown SB Liseth O Sharma B. Manganese dioxide nanoparticles protect cartilage from inflammation-induced oxidative stress. Biomaterials. (2019) 224:119467. 10.1016/j.biomaterials.2019.119467 31557589 PMC7025913

[90] Juan MW Xiong G Farooq U. Elements regulation during cartilage and bone deformity–potential clinical index in early diagnosis, monitoring and prognosis in children of kashin-beck disease. J Ayub Med Coll Abbottabad. (2015) 27:517–22. 26720997

[91] Rude RK Gruber HE Wei LY Frausto A Mills BG. Magnesium deficiency: effect on bone and mineral metabolism in the mouse. Calcif Tissue Int. (2003) 72:32–41. 10.1007/s00223-001-1091-1 12370796

[92] Ryu JH Yang S Shin Y Rhee J Chun CH Chun JS. Interleukin-6 plays an essential role in hypoxia-inducible factor 2α-induced experimental osteoarthritic cartilage destruction in mice. Arthritis Rheum. (2011) 63:2732–43. 10.1002/art.30451 21590680

[93] Tsao YT Shih YY Liu YA Liu YS Lee OK. Knockdown of SLC41A1 magnesium transporter promotes mineralization and attenuates magnesium inhibition during osteogenesis of mesenchymal stromal cells. Stem Cell Res Ther. (2017) 8:39. 10.1186/s13287-017-0497-2 28222767 PMC5320718

[94] Díaz-Tocados JM Herencia C Martínez-Moreno JM Montes de Oca A Rodríguez-Ortiz ME Vergara N. Magnesium chloride promotes osteogenesis through notch signaling activation and expansion of mesenchymal stem cells. Sci Rep. (2017) 7:7839. 10.1038/s41598-017-08379-y 28798480 PMC5552799

[95] Fairweather-Tait SJ Bao Y Broadley MR Collings R Ford D Hesketh JE. Selenium in human health and disease. Antioxid Redox Signal. (2011) 14:1337–83. 10.1089/ars.2010.3275 20812787

[96] Schoenmakers E Agostini M Mitchell C Schoenmakers N Papp L Rajanayagam O. Mutations in the selenocysteine insertion sequence-binding protein 2 gene lead to a multisystem selenoprotein deficiency disorder in humans. J Clin Invest. (2010) 120:4220–35. 10.1172/JCI43653 21084748 PMC2993594

[97] Andrade GRG Gorgulho B Lotufo PA Bensenor IM Marchioni DM. Dietary Selenium intake and subclinical hypothyroidism: a cross-sectional analysis of the ELSA-brasil study. Nutrients. (2018) 10:693. 10.3390/nu10060693 29848946 PMC6024881

[98] Zamamiri-Davis F Lu Y Thompson JT Prabhu KS Reddy PV Sordillo LM. Nuclear factor-kappaB mediates over-expression of cyclooxygenase-2 during activation of RAW 264. 7 macrophages in selenium deficiency. Free Rad Biol Med. (2002) 32:890–7. 10.1016/S0891-5849(02)00775-X 11978490

[99] Hozyen HF Khalil HMA Ghandour RA Al-Mokaddem AK Amer MS Azouz RA. Nano selenium protects against deltamethrin-induced reproductive toxicity in male rats. Toxicol Appl Pharmacol. (2020) 408:115274. 10.1016/j.taap.2020.115274 33038357

[100] Tang KK Li HQ Qu KC Fan RF. Selenium alleviates cadmium-induced inflammation and meat quality degradation via antioxidant and anti-inflammation in chicken breast muscles. Environ Sci Pollut Res Int. (2019) 26:23453–9. 10.1007/s11356-019-05675-0 31201704

[101] Wang C Wang H Luo J Hu Y Wei L Duan M. Selenium deficiency impairs host innate immune response and induces susceptibility to Listeria monocytogenes infection. BMC Immunol. (2009) 10:55. 10.1186/1471-2172-10-55 19852827 PMC2774297

[102] Zhang L Xia H Xia K Liu X Zhang X Dai J. Selenium regulation of the immune function of dendritic cells in mice through the ERK, Akt and RhoA/ROCK pathways. Biol Trace Elem Res. (2021) 199:3360–70. 10.1007/s12011-020-02449-5 33107016

[103] Scorei R Mitrut P Petrisor I Scorei I. A double-blind, placebo-controlled pilot study to evaluate the effect of calcium fructoborate on systemic inflammation and dyslipidemia markers for middle-aged people with primary osteoarthritis. Biol Trace Elem Res. (2011) 144:253–63. 10.1007/s12011-011-9083-0 21607703 PMC3241914

[104] Uluisik I Karakaya HC Koc A. The importance of boron in biological systems. J Trace Elem Med Biol. (2018) 45:156–62. 10.1016/j.jtemb.2017.10.008 29173473

[105] Afridi HI Kazi TG Brabazon D Naher S. Interaction between zinc, cadmium, and lead in scalp hair samples of Pakistani and Irish smokers rheumatoid arthritis subjects in relation to controls. Biol Trace Elem Res. (2012) 148:139–47. 10.1007/s12011-012-9352-6 22351104

[106] Alshenibr W Tashkandi MM Alsaqer SF Alkheriji Y Wise A Fulzele S. Anabolic role of lysyl oxidase like-2 in cartilage of knee and temporomandibular joints with osteoarthritis. Arthritis Res Ther. (2017) 19:179. 10.1186/s13075-017-1388-8 28764769 PMC5540418

[107] Opsahl W Zeronian H Ellison M Lewis D Rucker RB Riggins RS. Role of copper in collagen cross-linking and its influence on selected mechanical properties of chick bone and tendon. J Nutr. (1982) 112:708–16. 10.1093/jn/112.4.708 6121843

[108] Jonas J Burns J Abel EW Cresswell MJ Strain JJ Paterson CR. Impaired mechanical strength of bone in experimental copper deficiency. Ann Nutr Metab. (1993) 37:245–52. 10.1159/000177774 8311418

[109] Tiku ML Narla H Jain M Yalamanchili P. Glucosamine prevents *in vitro* collagen degradation in chondrocytes by inhibiting advanced lipoxidation reactions and protein oxidation. Arthritis Res Ther. (2007) 9:R76. 10.1186/ar2274 17686167 PMC2206377

[110] Blanco-Rojo R Pérez-Granados AM Toxqui L Zazo P de la Piedra C Vaquero MP. Relationship between vitamin D deficiency, bone remodelling and iron status in iron-deficient young women consuming an iron-fortified food. Eur J Nutr. (2013) 52:695–703. 10.1007/s00394-012-0375-8 22618893

[111] Kot K Kosik-Bogacka D Zietek P Karaczun M Ciosek Z Łanocha-Arendarczyk N. Impact of varied factors on iron, nickel, molybdenum and vanadium concentrations in the knee joint. Int J Environ Res Public Health. (2020) 17:813. 10.3390/ijerph17030813 32012969 PMC7038041

[112] Simão M Gavaia PJ Camacho A Porto G Pinto IJ Ea HK. Intracellular iron uptake is favored in Hfe-KO mouse primary chondrocytes mimicking an osteoarthritis-related phenotype. BioFactors (Oxford, England). (2019) 45:583–97. 10.1002/biof.1520 31132316

[113] Shmagel A Onizuka N Langsetmo L Vo T Foley R Ensrud K. Low magnesium intake is associated with increased knee pain in subjects with radiographic knee osteoarthritis: data from the Osteoarthritis Initiative. Osteoarthritis Cartilage. (2018) 26:651–8. 10.1016/j.joca.2018.02.002 29454594

[114] Chen Y Zhang Y Zhu YL Fu PL. Efficacy and safety of an intra-operative intra-articular magnesium/ropivacaine injection for pain control following total knee arthroplasty. J Int Med Res. (2012) 40:2032–40. 10.1177/030006051204000548 23206490

[115] Guo Y Zhou Y Yan S Qu C Wang L Guo X. Decreased expression of CHST-12, CHST-13, and UST in the proximal interphalangeal joint cartilage of school-age children with kashin-beck disease: an endemic osteoarthritis in china caused by selenium deficiency. Biol Trace Elem Res. (2019) 191:276–85. 10.1007/s12011-019-1642-9 30661165

[116] Ren FL Guo X Zhang RJ Wang Sh J Zuo H Zhang ZT. Effects of selenium and iodine deficiency on bone, cartilage growth plate and chondrocyte differentiation in two generations of rats. Osteoarthritis Cartilage. (2007) 15:1171–7. 10.1016/j.joca.2007.03.013 17490897

[117] Du B Zhou J Zhou J. Selenium status of children in Kashin-Beck disease endemic areas in Shaanxi, China: assessment with mercury. Environ Geochem Health. (2018) 40:903–13. 10.1007/s10653-017-0033-4 29018984

[118] Cao JJ Gregoire BR Zeng H. Selenium deficiency decreases antioxidative capacity and is detrimental to bone microarchitecture in mice. J Nutr. (2012) 142:1526–31. 10.3945/jn.111.157040 22739365

[119] Wang L Yin J Yang B Qu C Lei J Han J. Serious selenium deficiency in the serum of patients with kashin-beck disease and the effect of nano-selenium on their chondrocytes. Biol Trace Elem Res. (2020) 194:96–104. 10.1007/s12011-019-01759-7 31175635

[120] Jordan JM. An ongoing assessment of osteoarthritis in african americans and caucasians in North Carolina: the johnston county osteoarthritis project. Trans Am Clin Climatol Assoc. (2015) 126:77–86. 26330661 PMC4530702

[121] Hui W Young DA Rowan AD Xu X Cawston TE Proctor CJ. Oxidative changes and signalling pathways are pivotal in initiating age-related changes in articular cartilage. Ann Rheum Dis. (2016) 75:449–58. 10.1136/annrheumdis-2014-206295 25475114 PMC4752670

[122] Matsuzaki T Alvarez-Garcia O Mokuda S Nagira K Olmer M Gamini R. FoxO transcription factors modulate autophagy and proteoglycan 4 in cartilage homeostasis and osteoarthritis. Sci Transl Med. (2018) 10:eaan0746. 10.1126/scitranslmed.aan0746 29444976 PMC6204214

[123] Cornelis FMF Monteagudo S Guns LKA den Hollander W Nelissen R Storms L. ANP32A regulates ATM expression and prevents oxidative stress in cartilage, brain, and bone. Sci Transl Med. (2018) 10:eaar8426. 10.1126/scitranslmed.aar8426 30209244

[124] Huo R Yang L Zhang TG Wei JY. [Human selenium-containing single-chain variable fragment with glutathione peroxidase activity protects NIH3T3 fibroblast against oxidative damage]. Mol Biol. (2017) 51:483–9. 10.1134/S0026893317030074 28707665

[125] Chi Q Luan Y Zhang Y Hu X Li S. The regulatory effects of miR-138-5p on selenium deficiency-induced chondrocyte apoptosis are mediated by targeting SelM. Metallomics. (2019) 11:845–57. 10.1039/C9MT00006B 30869711

[126] Cheng AWM Bolognesi M Kraus VB. DIO2 modifies inflammatory responses in chondrocytes. Osteoarthritis Cartilage. (2012) 20:440–5. 10.1016/j.joca.2012.02.006 22353746 PMC3322270

[127] Ahmed HH Aglan HA Mabrouk M Abd-Rabou AA Beherei HH. Enhanced mesenchymal stem cell proliferation through complexation of selenium/titanium nanocomposites. J Mater Sci Mater Med. (2019) 30:24. 10.1007/s10856-019-6224-z 30747346

[128] Tare RS Howard D Pound JC Roach HI Oreffo RO. Tissue engineering strategies for cartilage generation–micromass and three dimensional cultures using human chondrocytes and a continuous cell line. Biochem Biophys Res Commun. (2005) 333:609–21. 10.1016/j.bbrc.2005.05.117 15946652

[129] Yan J Tian J Zheng Y Han Y Lu S. Selenium promotes proliferation of chondrogenic cell ATDC5 by increment of intracellular ATP content under serum deprivation. Cell Biochem Funct. (2012) 30:657–63. 10.1002/cbf.2845 22641559

[130] Yan J Fei Y Han Y Lu S. Selenoprotein O deficiencies suppress chondrogenic differentiation of ATDC5 cells. Cell Biol Int. (2016) 40:1033–40. 10.1002/cbin.10644 27425444

[131] Sun M Hussain S Hu Y Yan J Min Z Lan X. Maintenance of SOX9 stability and ECM homeostasis by selenium-sensitive PRMT5 in cartilage. Osteoarthritis Cartilage. (2019) 27:932–44. 10.1016/j.joca.2019.02.797 30858101

[132] Yan J Xu J Fei Y Jiang C Zhu W Han Y. TrxR2 deficiencies promote chondrogenic differentiation and induce apoptosis of chondrocytes through mitochondrial reactive oxygen species. Exp Cell Res. (2016) 344:67–75. 10.1016/j.yexcr.2016.04.014 27107686

[133] Huang TC Chang WT Hu YC Hsieh BS Cheng HL Yen JH. Zinc protects articular chondrocytes through changes in Nrf2-mediated antioxidants, cytokines and matrix metalloproteinases. Nutrients. (2018) 10:471. 10.3390/nu10040471 29641501 PMC5946256

[134] Aceituno-Valenzuela U Micol-Ponce R Ponce MR. Genome-wide analysis of CCHC-type zinc finger (ZCCHC) proteins in yeast, Arabidopsis, and humans. Cell Mol Life Sci. (2020) 77:3991–4014. 10.1007/s00018-020-03518-7 32303790 PMC11105112

[135] Wilhelmi V Fischer U Weighardt H Schulze-Osthoff K Nickel C Stahlmecke B. Zinc oxide nanoparticles induce necrosis and apoptosis in macrophages in a p47phox- and Nrf2-independent manner. PLoS ONE. (2013) 8:e65704. 10.1371/journal.pone.0065704 23755271 PMC3670863

[136] Rył A Miazgowski T Szylińska A Turoń-Skrzypińska A Jurewicz A Bohatyrewicz A. Bone health in aging men: does zinc and cuprum level matter? Biomolecules. (2021) 11:237. 10.3390/biom11020237 33567585 PMC7915903

[137] Mariani E Mangialasche F Feliziani FT Cecchetti R Malavolta M Bastiani P. Effects of zinc supplementation on antioxidant enzyme activities in healthy old subjects. Exp Gerontol. (2008) 43:445–51. 10.1016/j.exger.2007.10.012 18078731

[138] Hatakeyama D Kozawa O Otsuka T Shibata T Uematsu T. Zinc suppresses IL-6 synthesis by prostaglandin F2alpha in osteoblasts: inhibition of phospholipase C and phospholipase D. J Cell Biochem. (2002) 85:621–8. 10.1002/jcb.10166 11968002

[139] Rice JM Zweifach A Lynes MA. Metallothionein regulates intracellular zinc signaling during CD4(+) T cell activation. BMC Immunol. (2016) 17:13. 10.1186/s12865-016-0151-2 27251638 PMC4890327

[140] Khader A Arinzeh TL. Biodegradable zinc oxide composite scaffolds promote osteochondral differentiation of mesenchymal stem cells. Biotechnol Bioeng. (2020) 117:194–209. 10.1002/bit.27173 31544962

[141] Rodríguez JP Rosselot G. Effects of zinc on cell proliferation and proteoglycan characteristics of epiphyseal chondrocytes. J Cell Biochem. (2001) 82:501–11. 10.1002/jcb.1178 11500926

[142] Wang X Fosmire GJ Gay CV Leach RM. Jr. Short-term zinc deficiency inhibits chondrocyte proliferation and induces cell apoptosis in the epiphyseal growth plate of young chickens. J Nutr. (2002) 132:665–73. 10.1093/jn/132.4.665 11925458

[143] Weydert CJ Cullen JJ. Measurement of superoxide dismutase, catalase and glutathione peroxidase in cultured cells and tissue. Nat Protoc. (2010) 5:51–66. 10.1038/nprot.2009.197 20057381 PMC2830880

[144] Kloubert V Rink L. Zinc as a micronutrient and its preventive role of oxidative damage in cells. Food Funct. (2015) 6:3195–204. 10.1039/C5FO00630A 26286461

[145] Kwon JY Lee SH Jhun J Choi J Jung K Cho KH. The combination of probiotic complex, rosavin, and zinc improves pain and cartilage destruction in an osteoarthritis rat model. J Med Food. (2018) 21:364–71. 10.1089/jmf.2017.4034 29346012

[146] Sheikh AA Aggarwal A Aarif O. Effect of *in vitro* zinc supplementation on HSPs expression and Interleukin 10 production in heat treated peripheral blood mononuclear cells of transition Sahiwal and Karan Fries cows. J Therm Biol. (2016) 56:68–76. 10.1016/j.jtherbio.2016.01.002 26857979

[147] Müller RD John T Kohl B Oberholzer A Gust T Hostmann A. IL-10 overexpression differentially affects cartilage matrix gene expression in response to TNF-alpha in human articular chondrocytes *in vitro*. Cytokine. (2008) 44:377–85. 10.1016/j.cyto.2008.10.012 19026560

[148] Yan Z Qi W Zhan J Lin Z Lin J Xue X. Activating Nrf2 signalling alleviates osteoarthritis development by inhibiting inflammasome activation. J Cell Mol Med. (2020) 24:13046–57. 10.1111/jcmm.15905 32965793 PMC7701566

[149] Yang Y Wang Y Zhao M Jia H Li B Xing D. Tormentic acid inhibits IL-1β-induced chondrocyte apoptosis by activating the PI3K/Akt signaling pathway. Mol Med Rep. (2018) 17:4753–8. 10.3892/mmr.2018.8425 29328385

[150] Park C Hong SH Shin SS Lee DS Han MH Cha HJ. Activation of the Nrf2/HO-1 signaling pathway contributes to the protective effects of sargassum serratifolium extract against oxidative stress-induced DNA damage and apoptosis in SW1353 human chondrocytes. Int J Environ Res Public Health. (2018) 15:1173. 10.3390/ijerph15061173 29874784 PMC6025057

[151] Kim JH Jeon J Shin M Won Y Lee M Kwak JS. Regulation of the catabolic cascade in osteoarthritis by the zinc-ZIP8-MTF1 axis. Cell. (2014) 156:730–43. 10.1016/j.cell.2014.01.007 24529376

[152] Kuo CL Liu ST Chang YL Wu CC Huang SM. Zac1 regulates IL-11 expression in osteoarthritis. Oncotarget. (2018) 9:32478–95. 10.18632/oncotarget.25980 30197757 PMC6126702

[153] Fortier LA Schnabel LV Mohammed HO Mayr KG. Assessment of cartilage degradation effects of matrix metalloproteinase-13 in equine cartilage cocultured with synoviocytes. Am J Vet Res. (2007) 68:379–84. 10.2460/ajvr.68.4.379 17397292

[154] Carmona-Rivera C Carlucci PM Goel RR James E Brooks SR Rims C. Neutrophil extracellular traps mediate articular cartilage damage and enhance cartilage component immunogenicity in rheumatoid arthritis. JCI Insight. (2020) 5:e139388. 10.1172/jci.insight.139388 32484790 PMC7406272

